# Outbreak investigation of foot and mouth disease in Nangarhar province of war-torn Afghanistan, 2014

**DOI:** 10.1038/s41598-020-70489-x

**Published:** 2020-08-14

**Authors:** Abdul Wajid, Mamoona Chaudhry, Hamad Bin Rashid, Shakera Sadiq Gill, Sayed Rafiullah Halim

**Affiliations:** 1 Department of Epidemiology and Public Health, University of Veterinary and Animal Science, Abdul Qadir Jilani Road, Lahore, Pakistan; 2 Para Clinic Department, Veterinary Science Faculty, Nangarhar University, Jalalabad, Afghanistan; 3 Department of Clinical Medicine and Surgery, University of Veterinary and Animal Science, Lahore, Pakistan

**Keywords:** Pathogens, Virology

## Abstract

Afghanistan has long history of ongoing conflicts, resulting in massive destruction of the country’s infrastructure. Illegal trade of livestock between Afghanistan and Pakistan boosted the spread of Foot & Mouth Disease (FMD). Current study was conducted to investigate outbreaks of FMD occurred between April-August 2014 in Nangarhar, Afghanistan. Descriptive data about suspected FMD cases were collected from the Civil Veterinary Hospital, Nangarhar to analyze spatio-temporal pattern of FMD. Case farms (n = 137) were selected from list of clinically confirmed FMD outbreaks available in the hospital. Control farms (n = 137) were enrolled from neighboring premises of case farms. The epidemic curve showed that the virus is continuously circulating among susceptible population. The mean age of the oldest lesion was 2.8 days. Foot & Mouth Disease was more likely to occur in female animals compared to male animals (p < 0.001). Farmers having no ability to clinically recognize FMD (OR 5.8, 95% CI 1.4–23.8); previously having any FMD case in herd (OR 11.8, 95% CI 3.0–45.8), farms where animals leave shed during day (OR 15.4, 95% CI 5.6–42.0), and farms, where neighboring farmers used to visit the premises (OR 3.5, 95% CI 1.2–9.9) were identified as risk factors. Current findings may be used to create awareness of concerned veterinary health authorities about FMD control.

## Introduction

Foot & Mouth Disease (FMD) is a highly contagious, viral disease of cloven-hoofed domesticated (including cattle, water buffalos, sheep, and goat)^1^ and wild animals^2^. Foot & Mouth Disease is an economically expensive disease due to heavy losses to livestock industry with high morbidity in adult animals (especially cattle and pigs), decreased production efficiency and also mortality in young stock^3^. The FMD virus (FMDV) belongs to the genus *Aphthovirus* and family *Picornaviridae*^4^. The virus has seven major serotypes: O, A, C, Asia 1 and SAT 1 (South African Territory), SAT 2, and SAT 3^5^. These serotypes are immunologically unique and one serotype does not cross-protect against the others. However, continuous evolution of FMDV types have resulted into intra-serotypic subtypes that may cross-protect incompletely^6^. Continuous evolution of new isolates within a serotypes belonging to a particular geographic region make control of this disease difficult^7^.

Afghanistan was an agricultural country and exporter of livestock, but due to 40 years long ongoing conflicts and wars, its natural resources and trade has been damaged by military activities, refugees displacement, overexploitation of land and drought^8,9^. It is now an importer of meat and meat products and growing demand has increased profits for smuggling cattle to Afghanistan from Pakistan, with which it shares a long and porous border of 2,640 km^10–12^. Different type of agricultural and non-agricultural commodities are traded illegally at this border^13^. Nangarhar is considered as Afghanistan’s food basket due to its agriculture resources. About 70% of rural households, 64% of Kuchi nomads, and 18% of urban households in the province own livestock or poultry. The most commonly owned livestock are cattle (1–2 cows), donkeys, sheep, and goats. Different animal products like milk, meat, butter etc. is produced for household consumption while surplus is sold^14^. The buffalos are rarely present in herds^15^ and are usually imported from Pakistan for meat consumption^16^. The Nangarhar Province of Afghanistan is ecologically very similar to Pakistan and is connected through transhumance, trade and fattening enterprises^12,17^. Uncontrolled and Illegal movement of animals between two countries is linked to the enhanced transmission of trans-boundary animal diseases specifically FMD, which is endemic in both countries^12,18,19^. The seasonal movement of transhumant Kuchis tribes with their animals during winter also plays significant role in the spread of FMD regionally and globally^20,21^.

In Afghanistan and Pakistan serotypes O, A and Asia-1, are responsible for the outbreaks of FMD^19,22–24^. Continuous surveillance of virus transmission is essential to achieve better control of the disease in endemic countries. Worldwide strategy to respond in case of FMD is based on early detection and warning systems, prevention, and establishment of rapid response measures mechanism^25^.

Detailed epidemiological investigation of FMD outbreaks can give insight about disease patterns, which might be used for early warning and prospective control planning of the disease. Delayed case detection enhances disease transmission and epidemic risk, and subsequent economic losses in affected countries, which has been observed in previous FMD epidemics^26,27^. Comprehensive epidemiological knowledge of FMD is crucial for the development of efficacious surveillance and control programs^12,28^.

Foot & Mouth Disease is endemic in Afghanistan, however, epidemiological knowledge related to FMD is scarce in Afghanistan due to decades long war in the country, making many locations unsafe to visit, along with their geographical remoteness, which has hindered the delivery of effective veterinary services to the livestock sector by the Government and Non-Governmental Agencies (NGOs), and inefficient surveillance resulting in underreporting of FMD disease^12,29^.

Afghanistan was placed in stage 1 of OIE/FAO FMD-Progressive Control Pathway (FMD-PCP) by the GF-TADs FMD working group and regional advisory group for the West Eurasian region (7th Regional Progress Review Meeting, Bishkek, Kyrgyzstan, 2016, available at: https://www.fao.org/3/ca1257en/ca1257en.pdf). To reach at stage 2 of the FMD-PCP, it was advised to implement a risk-based strategic control plan and for that, investigation of outbreaks and identification of risk factors is very important.

Risk factors associated with FMD outbreaks have been identified through questionnaire based studies previously in several developing countries like Bhutan^1^, Thailand^30^, Japan^31^, Ecuador^32^, Bangladesh^33^, Cameroon^34^, Ethiopia^35^ and Sri Lanka^36^. Several risk factors were identified through these studies and among them were; animal transaction (buying, selling, or animal exchange between farmers), contact through animal movement between villages, free ranging of cattle herd and farm management practices. Animal husbandry practices e.g. exchange/sharing of farm utensils and services, sharing of breeding animals and movement of farm workers/personnel. Extensive published data is not available about the risk factors of FMD outbreaks in Afghanistan and to achieve the goal of FMD-PCP control strategy, a well-established knowledge of disease determinants is required. The present study was conducted to investigate the 2014 outbreaks of FMD in the war-torn areas of Behsud and Surkhrod Districts of Nangarhar Province, Afghanistan and to identify potential risk factors associated with these outbreaks.

## Results

### Epidemic curve of the FMD outbreaks

**Figure 1:**
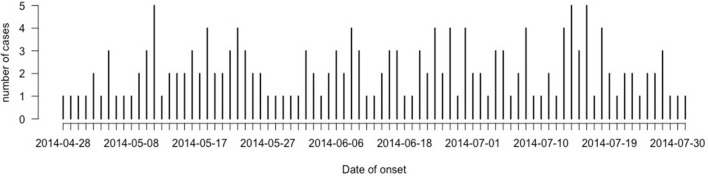
Epidemic curve of FMD outbreaks in Behsud and Surkhrod Districts, Nangarhar, Afghanistan April to July 2014.

**Figure 2:**
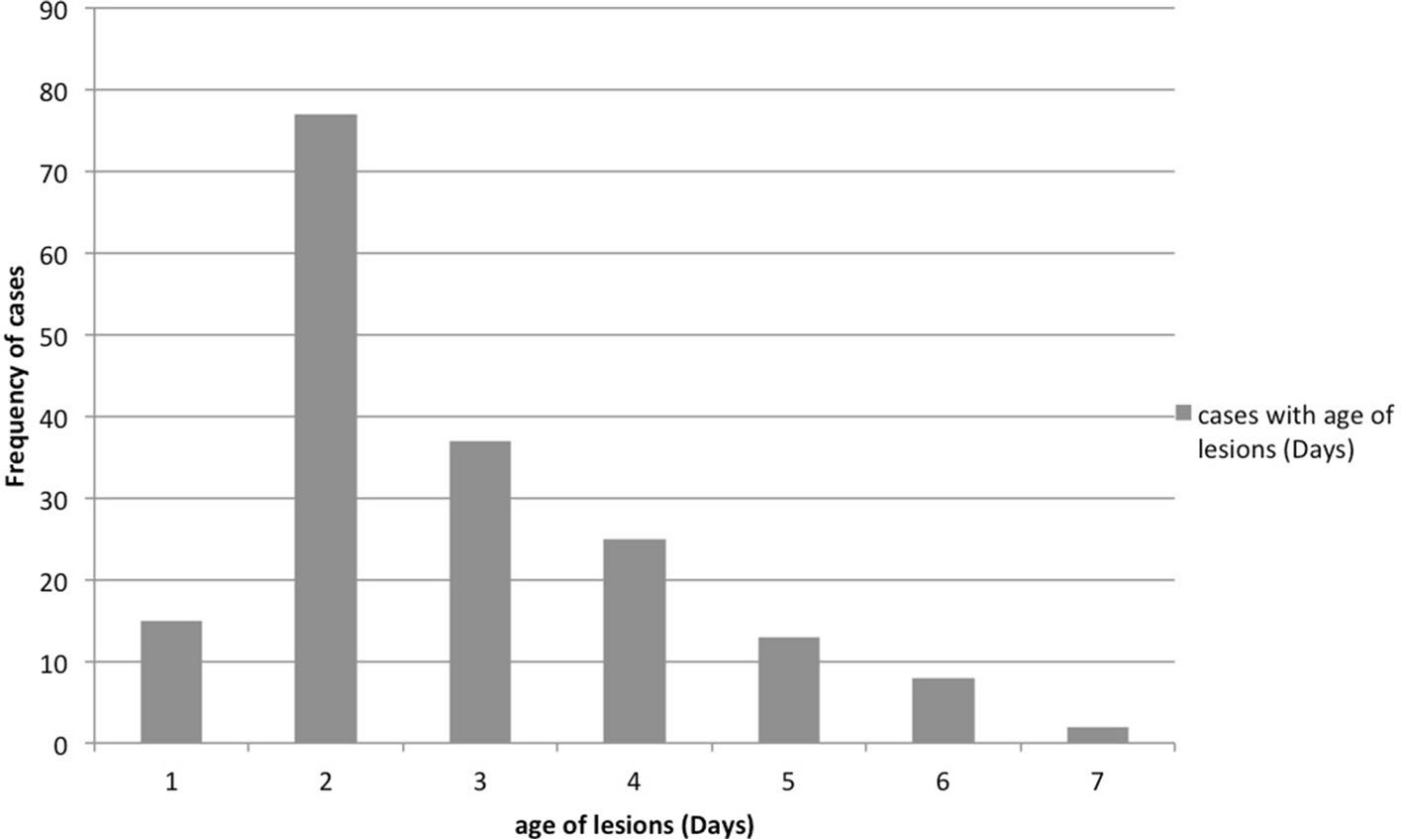
Distribution of the age of oldest lesion in the FMD outbreak/cases.

Data were used from all infected premises from where animals were brought for clinical examination at CVH, Nangarhar (n = 177). The epidemic curve showed a pattern of propagated epidemic (CDC, 2012). It showed that the virus is spreading persistently from infected to susceptible population (Fig. 1). The mean age of the oldest lesion was 2.8 d [standard deviation (s) = 1.3], with a minimum age of 1 day and a maximum of 7 days on infected farm (Fig. 2). Proportion of premises with female FMD positive animals was higher (n = 132, 74.6%), as compared to premises with infected male animals (n = 45, 25.4%). Foot & Mouth Disease was more likely to occur in female animals as compared to male animals (χ^2^ = 42.8; p < 0.001). Majority of farmers (n = 78, 44%) reported that FMD was observed in their herds once in a year.

### Spatial and temporal patterns of FMD outbreaks

**Figure 3:**
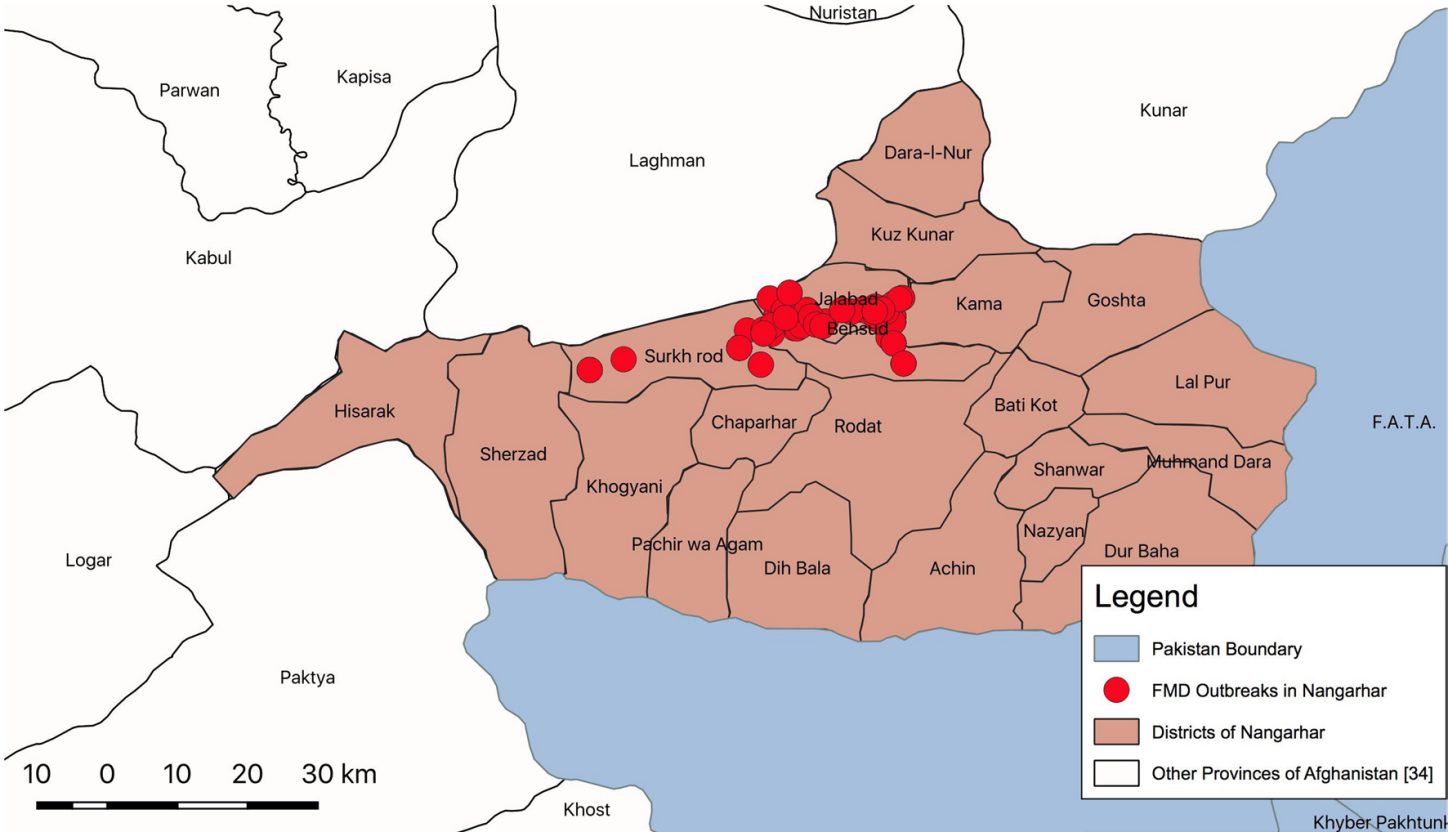
Outbreak map of FMD cases in Nangarhar Province. Map was created using QGIS (https://qgis.org/en/site/) by the senior author (MC).

**Figure 4:**
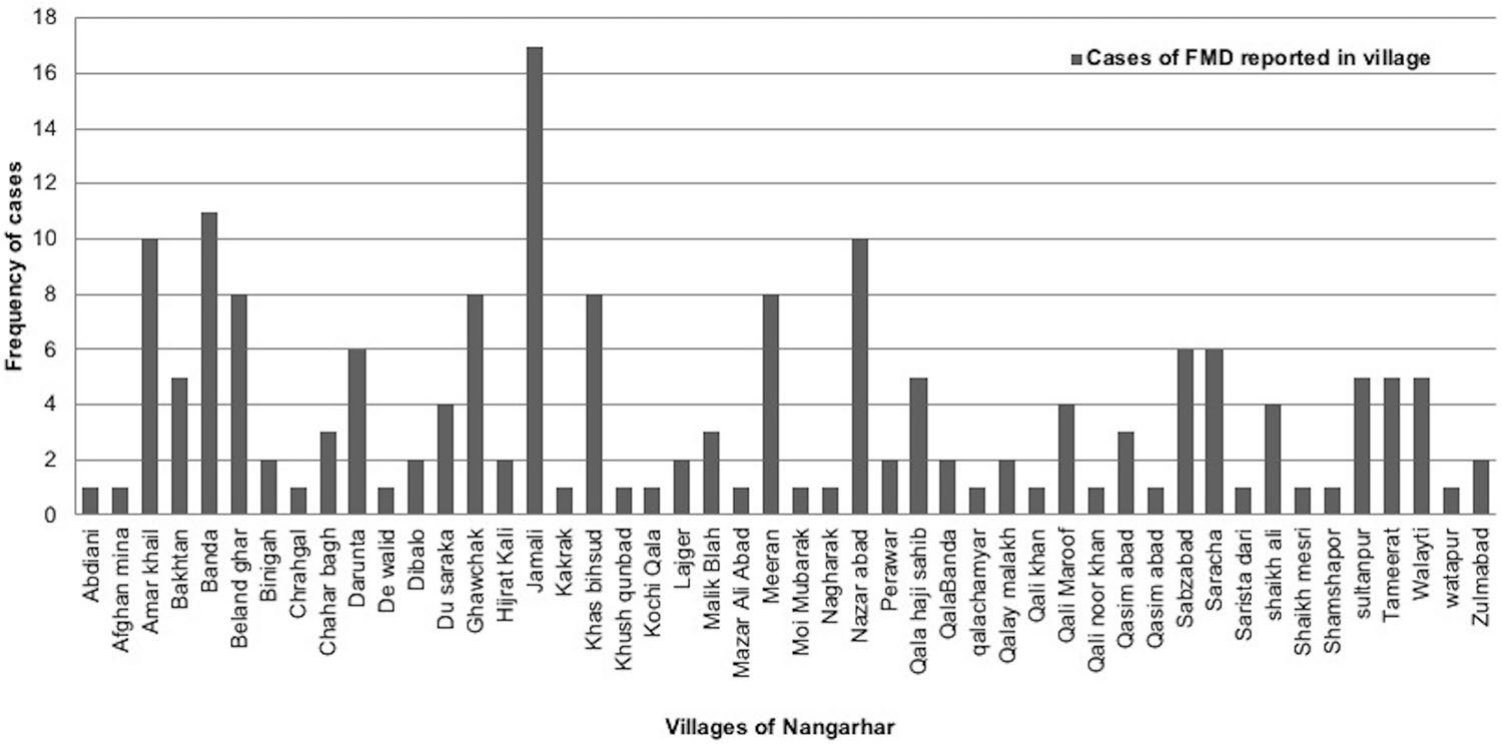
Distribution of FMD outbreaks/cases in different villages of Nangarhar, Afghanistan.

Outbreaks were spatially distributed in study area (Fig. 3). During temporal analysis of outbreak data, it was recorded that the maximum outbreaks (n = 63, 35.6%) occurred in July 2014 and in the Behsud District (n = 92). Maximum number of outbreaks (n = 17) occurred in Jamali village of Behsud District (Fig. 4) and the most affected species was sheep (n = 88, 49.7%), followed by cattle (n = 80, 45.2%) and goat (n = 9, 5.1%). The male:female ratio for ovine was 9 to 20.3, for bovine it was 3:17 and for caprine was 2:1. Average herd size was 10 (range between 1–82) animals.

### Case–control study

A total of 274 farmers (137 case farms, 137 control farms) were interviewed from the study area. Most of the farmers (n = 233, 85%) were rural smallholder farmers, 6.6% (n = 18) were rural commercial farmers and 8.4% (n = 23) were peri-urban commercial farmers. Average number of cattle in herd was 4 (range 0–20, there were 30 premises where no cattle was present), while average number of sheep was 2 (range 0–39, sheep were not present in the herd at 190 premises) and goats was 4 (range 0–60, no goat was present in herd at 106 premises) respectively. Most of the farmers (n = 74) possessed one acre of land. Out of 37 risk factors, 14 variables were selected for inclusion in multivariable analysis based on biological plausibility and selection criterion (Table 1). Nine factors having p > 0.25 were excluded from further analysis. Logistic regression analysis of 7 variables could not be conducted due to zero cell values in 2 × 2 contingency tables. Six variables were correlated [rho (ρ) ≥ 0.5] with others variables and from each pair of correlated variables only biologically plausible variables were retained in analysis. Two variables were excluded due to insufficient discordant pairs. In the final multivariable model, four variables were identified as significantly associated with the FMD outbreak (Table 2). Case farms were more likely to have farmers with no ability (unable to distinguish visible FMD lesion in their animals) to clinically diagnose FMD (OR: 5.8, 95% CI: 1.4–23.8, p < 0.05) compared to control farms. The odds of previously having any case of FMD in herd was 11.8 times more in case farms (95% CI 3.0–45.8, p < 0.001) when compared to exposure in control farms. Similarly, case farms where animals leave shed during day (usually for grazing outside the premises) were more likely to have FMD outbreaks (OR 15.4, 95% CI 5.6–42.0, p < 0.001) when compared to control farms. The case farms, where neighboring farmers used to visit the premises were 3.5 times more likely to have FMD (95% CI 1.2–9.9, p < 0.05).

**Table 1:** Univariable analysis of potential risk factors for presence of FMD in two districts of Nangarhar, Afghanistan (variables included in modeling).

Sr. no	Variable	Response level	Control (FMD negative)	Case (FMD positive)	OR	95% CI	p-value
1	Premises	Rural small holder	125	108	Reference		–
		Rural commercial	4	14	3.6	1.2–11.2	0.022
		Peri-urban commercial	8	15	2.0	0.83–4.74	0.119
2	Livestock farming experience	1–10 year	47	53	Reference		–
		11–20 year	62	22	0.3	0.2–0.6	< 0.001
		21–30 year	14	27	2.2	0.9–5.0	0.063
		31–40 year	6	12	2.0	0.7–5.8	0.208
		41–50 year	6	13	2.4	0.8–7.6	0.130
		More than 50 years	2	10	6.1	1.2–29.7	0.025
3	Sex of animal	Male	18	33	Reference		0.016
		Female	119	104	0.4	0.2–0.9	
4	Goat owned	No	75	31	Reference		< 0.001
		Yes	62	106	3.9	2.2–6.9	
5	Sheep day management	Always housed in shed/pens	119	88	Reference		< 0.001
		Not always housed in shed/pens	18	49	3.2	1.8–5.9	
6	Feeding concentrate	Oil seed cake	73	88	Reference		–
		Oil seed cake/bread & wanda	15	10	0.6	0.3–1.4	0.247
		Wanda	49	39	0.7	0.4–1.2	0.167
7	Animal share manger/trough	No	86	31	Reference		–
		Yes	51	106	3.9	2.4–6.4	< 0.001
8	Animal leave shed/premises during day	No	121	25	Reference		–
		Yes	16	112	20.2	8.2–49.6	< 0.001
9	Use of dung as fuel/manure	No	15	9	Reference		–
		Yes	122	128	1.8	0.7–4.2	0.207
10	Neighboring farmers visiting your premises	No	96	57	Reference		–
		Yes	41	80	3.2	1.9–5.4	< 0.001
11	Recently purchased animals	No	100	90	Reference		–
		Yes	37	47	1.4	0.8–2.4	0.184
12	Ability to clinically recognize FMD	Yes	115	104	Reference		–
		No	22	33	1.6	0.9–3.0	0.105
13	Having any case of FMD in herd previously	No	103	48	Reference		–
		Yes	34	89	4.9	2.8–8.8	< 0.001
14	Any animal die in the last outbreak	No	127	100	Reference		–
		Yes	10	37	6.4	2.5–16.4	< 0.001

**Table 2:** Potential risk factors for presence of FMD in two districts of Nangarhar, Afghanistan in final model in multivariable analysis.

Sr. no	Variable	Response level	Regression coefficient	Standard error	OR	95% CI	p-value
1	Animal leave shed/premises during day	No			Reference		–
		Yes	2.73	0.51	15.4	5.6–42.0	< 0.001
2	Having any case of FMD in herd previously	No			Reference		–
		Yes	2.46	0.69	11.8	3.0–45.8	< 0.001
3	Neighboring farmers visiting your premises	No			Reference		–
		Yes	1.25	0.53	3.5	1.2–9.9	0.018
4	Ability to clinically diagnose FMD	Yes			Reference		–
		No	1.76	0.71	5.8	1.4–23.8	0.014

R^2^ = 0.387 (out of possible 0.5).

## Discussion

In Afghanistan and Pakistan, FMD is the main trans-boundary endemic disease and has the ability to spread very rapidly crossing national and international borders, causing serious economic losses and have affected food security and national economics^18,19^. Due to continuous war and ongoing conflict in the region, very limited data is available about epidemiology of FMD in Afghanistan^12^. The current study investigated outbreaks of FMD and potential risk factors associated with these outbreaks in Behsud and Surkhrod Districts, Nangarhar Province, Afghanistan, to present the glimpse of epidemiological situation of disease in the country. A total of 177 outbreaks were reported by civil veterinary hospitals in two districts of the province. The propagated pattern of epidemic curve of FMD outbreaks in study area could be attributed to high density of susceptible livestock population (Cattle, Sheep and Goats) and poor biosecurity in these rural smallholder herds and villages. Mixing of diseased and healthy animals while grazing at pastures escalate the probability of exposure to circulating viruses and enhance disease spread^37^. It showed that the virus is spreading persistently from infected to susceptible population.

The outbreak map identified several villages in Behsud District as clusters of FMD with maximum number (n = 17) of outbreak reported in Jamali village during the study period. Behsud District has high density of livestock compared to Surkhrod District^38^. Livestock density can impact transmission dynamics of FMD and would require additional strategies to control disease in highly dense areas compared to low dense areas^37^.

The number of outbreaks accelerated in May, June and July 2014, corresponding to the increased demand and transportation of animals to regional markets during Ramadan and at the eve of Eid-ul-Fitar (Muslim celebration event) (June–July, 2014). Gunasekera, et al.^36^ reported escalated number of outbreaks due to peak movement of animals for the festival celebrations for Muslim and Buddhist community. Osmani, et al.^12^ also reported that there is high probability of spread of FMD in the region through the continued movement of refugees across the border between Afghanistan and Pakistan and significant legal and illegal movement of animals especially at the religious occasion such as Eid-ul-Adha. Animal movements have been recognized as one of the most common method of transmission of FMD^1,37,39,40^. As Nangarhar province share border with Pakistan, seasonal migrations of nomadic populations through transhumance routes between Pakistan and Afghanistan along with their livestock also poses risk of trans-boundary diseases. The illegal movement of animals across the borders and two-way trade of livestock remained a major problem in terms of disease management including FMDV^19,41–43^.

Out of 177 positive cases 25.4% were male and 74.6% were female, which has been reported previously^44^. In current study maximum number of FMD cases were reported on the day when the lesion age was approximately 2 days (n = 77, 43.5%). Previously the mean age of oldest lesion was reported as 1.80 day^27^.

Small ruminants play a crucial role in the epidemiology of FMD transmission as they often show inapparent infection in these hosts and are largely ignored during national vaccination programs^39,45,46^. Furthermore, these species have ability to become carriers and can act as reservoir for further infection and spread of disease, hence trade of live sheep and goats present a major risk of entry of FMD to disease-free countries^47^. In current study, small ruminants, both sheep (n = 88) and goat (n = 9) were reported to be infected with FMD, along with cattle (n = 80). Sheep are highly susceptible to virus infection and can excrete virus through aerosol route and have been reported for transmission of FMDV within countries and across borders^46^. The FMDV can persist in sheep for up to 12 months and in goats for 2–3 months^48^. Nearly every herd in Afghanistan has a small ruminants animal (sheep or goat), which put them at higher risk of remained as carrier and being ignored due to subclinical nature of disease in small ruminants^47^. Most of the farmers (44%) reported that the FMD occurred on their premises once in a year. Similar results were documented previously^49^.

In current study four risk factors were identified. A farmer with no ability to clinically recognize FMD was 5.8 times more likely to have positive farm. Ability to recognize FMD clinically enable farmers to report disease promptly and early detection eventually lead to rapid response and better management of cases at premises^27^. Given the potential of FMD for rapid spread, it is essential that suspected cases are quickly reported and investigated by means of rapid and accurate tests, so that control measures can be speedily implemented^43^.

Previously having any case of FMD in herd increased the likelihood of being a case farm 11.8 times (95% CI 3.0–45.8, p < 0.001) compared to control farms with no previous FMD case in herd. Presences of any previous case in herd or any infected premises nearby keep farmers alert and more vigilant about clinical signs. They interact with veterinary staff more frequently than others to seek help for existing cases. These visits and interaction improve the farmer’s awareness about the disease and hence increase the likelihood of early detection of any new case in the herd, subsequently helping veterinary authorities to fully understand the extent of FMD infection in livestock and identifying any arising outbreak^27,50^.

In this study, case farms where animals leave shed during day (usually for grazing outside the premises) were more likely to have FMD outbreaks (OR 15.4, 95% CI 5.6–42.0). Susceptible animal are usually exposed to large quantity of viruses shed by infected animals at a grazing area, which can spread disease to non-infected animals^35,36,43,51^.

Farms having frequent visits by the neighboring farmers were more likely to have FMD than those farms where access to premises was restricted (OR 3.5, 95% CI 1.2–9.9). Lack of biosecurity has been recognized as a major factor in the spread of FMD^1,36,50,52^.

## Conclusion

Our findings have provided an overview of the FMD outbreaks in Nangarhar Province of Afghanistan and have identified risk factors for the occurrence of FMD outbreaks. These finding suggests continuous circulation of FMD virus in livestock of Afghanistan. It may pose a serious threat to the livestock industry of the country and to the global food security. The current findings could be used to generate suitable recommendations for control of FMD in the country. To achieve the goals of Progressive Control Pathway for FMD control, risk factors identified from this study may be included in the FMD control plan for Afghanistan. For future research, longitudinal and surveillance studies are recommended to monitor the virus circulation and disease dynamics.

Our study has several limitations due to unavoidable circumstances. Scarcity of the data due to continuous war and conflict in the region, resulting in massive damage to the veterinary infrastructure, was a major hindrance in collection and collation of data. As very limited veterinary health facilities were available in the area, very few outbreaks were reported to the veterinary authorities, which might have introduced reporting bias in data. A clinical diagnostic criterion based of clinical sign was used by the veterinarian to diagnose case and control farms due to unavailability of confirmatory diagnostic tests. This selection criterion might have led to misclassification bias especially when selecting control farms from an FMD endemic area ^36^. As case–control study was based on questionnaire data, recall bias might have encountered in data. Furthermore, exact geographical coordinate where the outbreak occurred, were not available, thus only the available village locations were considered in each district to develop distribution map.

## Methods

### Study area

**Figure 5:**
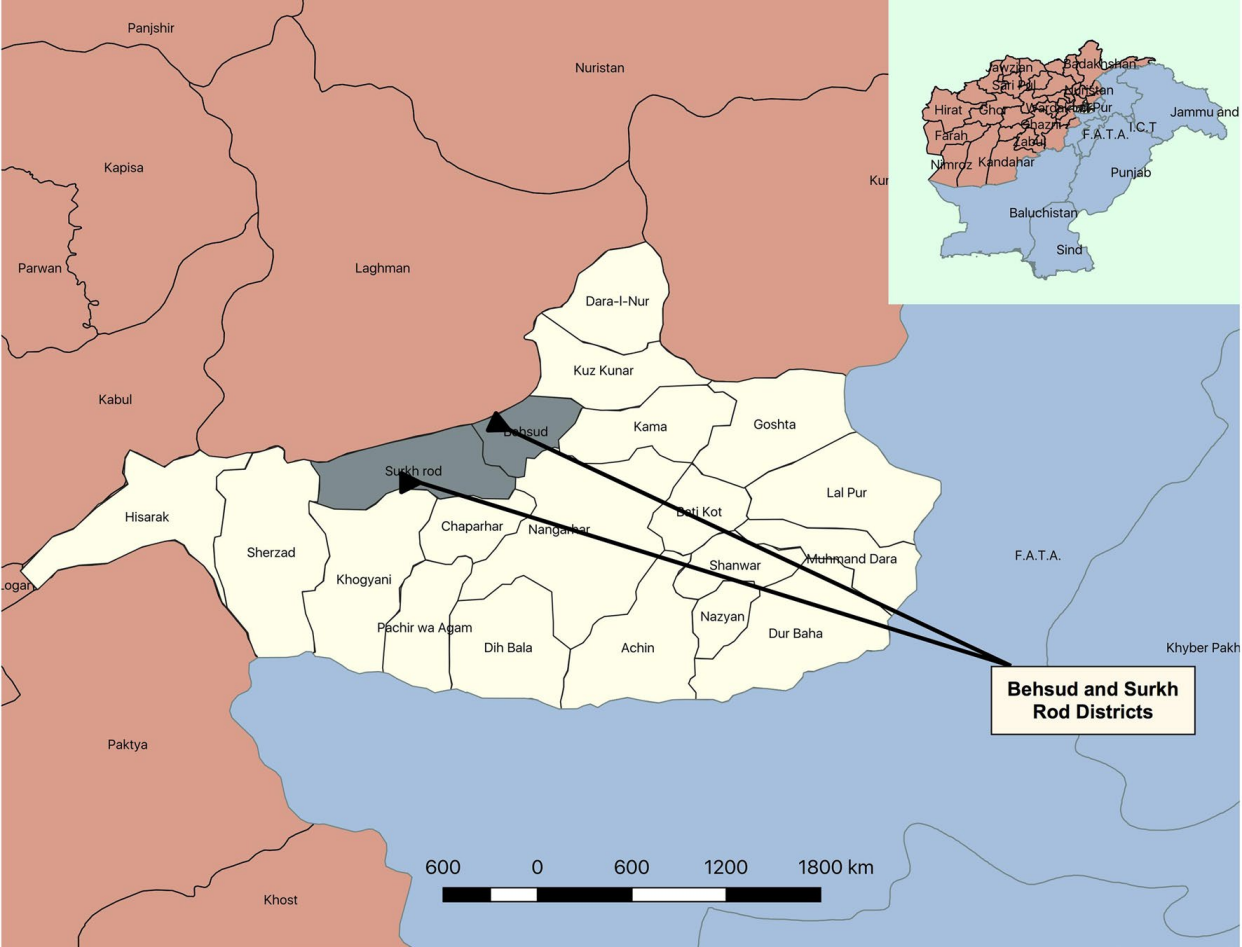
Map showing selected districts of Nangarhar, Afghanistan. Map was created using QGIS (https://qgis.org/en/site/) by the senior author (MC).

The outbreak investigation was carried out in two districts (Behsud and Surkhrod) of the Nangarhar Province of Afghanistan (Fig. 5). Located at 34°10′ N latitude and 70°37′ E longitude in east of Afghanistan, Nangarhar shares a border with Khyber Pakhtunkhawa province of Pakistan. Pashtun are the major ethnic group of population on both side of the boundary. Jalalabad, the capital city of Nangarhar, is situated on an ancient trade route connecting Kabul to Peshawar and the Indian sub-continent via Kyber Pass^53^.

### Study design

In veterinary medicine, plenty of published information is available about FMD outbreak investigation^35,39,54–56^. For current study, outbreak investigation was conducted following ten steps of outbreak investigation^57^. All study protocols including humans were approved by Institutional Review Committee for Biomedical Research, University of Veterinary and Animal Sciences, Lahore, Pakistan. There was no experimentation on human and animal subjects and only data was collected from the farmers. Local veterinary authorities in Afghanistan were contacted to seek permission to contact owners of animals from selected premises. In person contact was made to reach owners and objective of the study were explained and they were then asked to participate in the study. After informed consent from owner, a face-to-face interview was conducted to collect information about risk factors using a pre-structured questionnaire. Most farmers were reluctant to provide any written consent due to ongoing war and privacy. Hence, only those farmers, who were willing to participate in the study, provided data about outbreaks of FMD on their premises.

The methods in current study were carried out in accordance with relevant guidelines and regulations to collect observational data as given in Declaration of Helsinki.

### FMD outbreak analysis

#### Outbreak definition

The current study was conducted to investigate the outbreaks of FMD in study area. According to National Livestock Census report of Afghanistan, the total number of cattle in the country is 3.7 million, number of sheep is 8.8 million, and number of goat is 7.3 million^38^. Exact data about the livestock population and composition in Nangarhar Province was not available. It is estimated that almost 70% of the households in the rural areas of Nangarhar keep one or two cows^53^. For the current study, an outbreak of FMD was declared if one or more clinical cases of FMD occurred in a herd during the same time period (incubation period). If a case occurred in a herd or farm, which was separated from other herd or farm by physical barriers such as rivers, streams, hills or mountains or by time barrier i.e. occurring in same farm or herd but at different time period, it was considered a separate outbreak. For outbreak investigation, an animal was considered positive for FMD based on the clinical definition i.e. high body temperature, excessive salivation, vesicles formation on the tongue, nose, lips, oral mucosa, plus the inter-digital spaces and coronary bands on the feet, along with few or all signs of reduced appetite, depression, fever, hypersalivation, and lameness^36,58–60^.

### Data source for outbreak analysis

An outbreak was confirmed by the veterinary officer of the reporting Civil Veterinary Hospitals (CVH) based on clinical diagnosis in suspected animals attending CVH from the farms in the selected districts. Serological or molecular diagnosis of the samples was not available due to poor diagnostic facilities resulting from decades long war and conflict in Nangarhar. In Kabul, Central Veterinary Diagnostic and Research Laboratory (CVDRL) has facilities to detect and genotype FMDV, however, continuous conflict and budget constraints caused shortage of supplies and reagents in the country and laboratory heavily relies upon the financial aid of international organizations, such as the Food and Agricultural Organization of the United Nations (UNFAO), to support the diagnosis of the disease in its livestock^12^. As a result, diagnostic testing remained infrequent in the country.

All reported outbreaks/diseased animals during the study period were included in the analysis.

Data about these outbreaks from April to August 2014 were retrieved from the CVH of Nangarhar, Afghanistan on a predesigned questionnaire. Data about number of diseased animals, age of lesions, infection date, date of reporting, geographical location (village, district), species of infected animal, herd size, sex and age of animal were collected and analyzed.

### Case control study to identify risk factors of FMD outbreaks

### Case and control farm selection

The study was done in areas frequently experiencing FMD outbreaks in two districts (Behsud, & Surkhrod) of Nangarhar Province. The eligible population was all farm premises having cloven-footed animals in villages of both districts in Nangarhar, Afghanistan. The final study population was farm premises whose animals attended CVH, Nangarhar, for treatment purpose. A case farm was defined as a farm premises having animals (cattle/buffalo/sheep/goat) with clinical signs or lesions characteristics of FMD (clinically diagnosed cases) with or without laboratory confirmatory diagnosis^58^ reported in the last 4 months to the CVH, Nangarhar. Control farm was defined as a farm with animal negative for FMD infection based on herd history and lack of clinical signs of FMD in the last 4 months. Control farms were selected at random from the surrounding neighborhood of case farms in the same village. The owner of the control farms was requested to participate in the study and after formal consent; data were collected from that farm. Each case farm was matched with the control farm on geographical area i.e. same village. Due to unavailability of funds and diagnostic facilities to test the disease or health status of animals at each case and control farms, only the clinical records of the animals at farms were considered for selection. The FMD status of selected case farms was confirmed on the information available in the records of CVH of the area. The trained veterinarian diagnosed the animals, based on clinical signs and recorded the data in hospital registry^36^.

### Sample size for case–control

The sample size was computed to detect an odds ratio of > 2.0 with 95% confidence interval with 80% power and assuming that 33% of controls are exposed to FMDV. The minimum sample size required to conduct study was 274 samples (137 case farms and 137 control farms) with a case–control ratio of 1:1. The sample size calculation was done using WINPEPI software (Version 11.17. 2012)^61^. The case farms were selected from list of farm premises (N = 177) with confirmed status of FMD (outbreak data) and farmers were invited to participate. If farm owner, refused to participate, next farmer on the list was approached. Control farms were selected from the neighboring farm premises of these case farms with no history of FMD in past 4 months.

### Questionnaire for case–control study

A predesigned questionnaire tested with 10 farmers in a pilot study, was used to retrieve information about risk factors of case and control farms. The questionnaire contained 37 questions about biologically plausible risk factors. The questionnaire was prepared after literature review of various studies conducted to identify risk factors in various parts of the world^31,35,54,62^. Data were collected in a face-to face interview with farm owner about demographic characteristics of farmer, socioeconomic status, feeding practices, biosecurity measures and other relevant factors (Annexure I). The questionnaire was administered in local language in a face-to-face interview with the farm owner, who was willing to participate in the study and fulfilled the selection criteria.

### Statistical analysis

Epidemiological and temporal analyses were performed in R software (version 3.2.3)^63^. The frequency distribution of diseased animals by age group of animals, sex of animal, area of residence (District & Village), age of lesions, frequency of occurrence of FMD in same herd/animal, species affected, season and reporting date was calculated by using epiDisplay package in R software^64^. Chi-Square test was used to estimate any association among various characteristics. The epidemic curve was drawn to observe the dynamics of epidemic. We performed a logistic regression analysis using presence of clinical signs as the outcome of interest. All biologically plausible risk factors were screened in univariable analysis by using survival package (version, 2.37.7.0) in R software^65^. Predefined criteria i.e. p < 0.25, was used to select variables for inclusion in a multivariable model^66^. Significant variables at p < 0.25 in the univariable analysis were included in multivariable analysis. Collinearity of variables was tested by using R package ellipse^67^. A multivariable model was derived by forward stepwise selection procedure, following selection criteria i.e. to remove the variable with p > 0.25^68^. Variables with p ≤ 0.05 based on Wald Statistic (or log likelihood ratio test for categorical variables with 3 or more levels) were retained in the model. To estimate the strength of association, Odds ratios (ORs) and corresponding 95% confidence intervals (CIs) were calculated^66^.

### Spatial analysis of FMD outbreaks

QGIS-2.14.3 (available at https://www.qgis.org/en/site/forusers/download.html#) was used to visualize the spatial distributions of diseased animals in study area. The coordinates of villages with outbreak were spatially retrieved via MapCarta (available at https://mapcarta.com). The village location records were obtained from CVH Nangarhar as provided by the farmers. A dot map for FMD outbreaks was created using QGIS (available at https://qgis.org/en/site/).

## Supplementary information

Supplementary Information.

## Data Availability

The datasets generated during and/or analyzed during the current study are available from the corresponding author on reasonable request.
