# Supplementary material for: Outbreak investigation of foot and mouth disease in Nangarhar province of war-torn Afghanistan, 2014
Source: Sci Rep. 2020 Aug 14;10:13800. doi: 10.1038/s41598-020-70489-x (PMC7429494; doi:10.1038/s41598-020-70489-x)
Supplement: Supplementary file 1 — Supplementary Information. [file 41598_2020_70489_MOESM1_ESM.docx]

**Outbreak Investigation of Foot and Mouth Disease in Nangarhar Province of War-Torn Afghanistan, 2014.**

**Authors:** Abdul Wajid,^1,2*^, Mamoona Chaudhry^1*^, Hamad Bin Rashid^3^, Shakera Sadiq Gill^1^, Sayed Rafiullah Halim^2^

**Affiliations:**

^1^Department of Epidemiology and Public Health, University of Veterinary and Animal science, Lahore.

^2^Para Clinic Department, Veterinary Science Faculty, Nangarhar University, Afghanistan.

^3^Department of Clinical Medicine & Surgery, University of Veterinary and Animal science, Lahore.

- **This section must be filled in before completing the questionnaire:**

| Date: | Questionnaire No.: |
| --- | --- |

| Province: | District: |
| --- | --- |
| Village/Town: | |
| Name of Respondent: | Sex: **Male □ Female □** |
| Farm Address: | |

- **Tick only one answer:**

**Farmer’s Profile**

1. Premises/farm type: ……...........................................................................................

………………..**rural smallholder □ rural commercial □ Peri-urban commercial □**

2. Herd size ............................................. **1-10 animals □ 11-20 animals □ 21-above□**

3. Qualification of farmers...................................................................................

..............................**literate (can read & write) □ illiterate (can not read & write) □**

4. How long have you been engaged with livestock farming ………______________

5. Purpose of keeping livestock: ................................................ **for** **meat □ for milk □**

6. Sex of animal............................................................................... **Male** □ **Female** □

**Livestock animals raised by the farmer**

7. Cattle: .............. **Yes** □ **No** □ 8. If yes, number .... ________________________

9. Sheep: ............. **Yes** □ **No** □ 10. If yes, number .... ________________________

11. Goat: ............... **Yes** □ **No** □ 12. If yes, number .... ________________________

13. Total no. of animals: .............................................. ________________________

**Management Practices**

How are the animals housed during the day-time: ………………………….…………

|  | Animal type | Housing type |
| --- | --- | --- |
| 14 | Cattle | **Always housed in sheds/pens □ Not always housed in sheds/pens □** |
| 15 | Sheep | **Always housed in sheds/pens □ Not always housed in sheds/pens □** |
| 16 | Goat | **Always housed in sheds/pens □ Not always housed in sheds/pens □** |

How are the animals housed during the night-time: …….……………………..………

|  | Animal type | Housing type |
| --- | --- | --- |
| 17 | Cattle | **Always housed in sheds/pens □ Not always housed in sheds/pens □** |
| 18 | Sheep | **Always housed in sheds/pens □ Not always housed in sheds/pens □** |
| 19 | Goat | **Always housed in sheds/pens □ Not always housed in sheds/pens □** |

20. What do you feed your livestock: .................................................................... ………. **Seasonal green fodder □ concentrate □ Green Fodder & Wheat straw □**

**Seasonal green fodder/Wheat straw & concentrate □ Others □**

21. If you feed concentrate, what do you use: ......................................................... ………………...…..… **Oil seed cake □ Oil seed cake/ bread & wanda □ Wanda □**

22. Do your animal share the manger/trough with other animals ………………………………………………………………..............…… **Yes □ No □**

23. Do your animals drink water from water channels: …….......................**Yes □ No □**

24. Do your animals leave the shed/farm premises during the day: ……... **Yes □ No □**

25. If “Yes” to question no. 24, do your animals mix with animals from other premises …………………………………………………………………... **Yes □ No □**

26. Do you use dung of your animals as fuel/manure: ............................... **Yes □ No □**

27. Do your neighbouring/other farmers visit your premises: .................... …………………………………………………………...……….……..… **Yes □ No □**

28. Have you purchased any animal recently (less than two weeks)……... **Yes □ No □**

29. If “Yes” from where did you purchase/acquire the animal…………**Neighbouring premises** **□ Auction Market □ Production farm □ Other □**

30. Can you clinically recognize FMD: ……...……………….….…….. **Yes □ No □**

31. Have you had any case of FMD in your herd previously: ………..…… ………………………………………………….…………………………. **Yes □ No □**

32. Has your village/area had any case of FMD in animals previously: ……………………………………….……………………………………. **Yes □ No □**

33. If “Yes”, when the last outbreak occurred: ………………………………...……..

....................................................................... **15 days ago** **□ more than 15 days ago** **□**

34. How many animals were infected in the last outbreak: ……………………..…...

………………..........................................…. **All □ Half □ Few □ Don’t remember □**

35. Did any animal/animals die in the last outbreak: ………………...….. **Yes □ No □**

36. Did you cull/sell any animal/animals in the last outbreak: ……....….. **Yes □ No □**

37. Is there any Veterinary hospital/dispensary near your premises: .......... **Yes □ No □**
